# Chinese Clinical Named Entity Recognition in Electronic Medical Records: Development of a Lattice Long Short-Term Memory Model With Contextualized Character Representations

**DOI:** 10.2196/19848

**Published:** 2020-09-04

**Authors:** Yongbin Li, Xiaohua Wang, Linhu Hui, Liping Zou, Hongjin Li, Luo Xu, Weihai Liu

**Affiliations:** 1 School of Medical Information Engineering Zunyi Medical University Zunyi China; 2 Radiology Department Beilun District People’s Hospital Ningbo China

**Keywords:** clinical named entity recognition, ELMo, lattice LSTM, deep learning, neural network, sequence tagging

## Abstract

**Background:**

Clinical named entity recognition (CNER), whose goal is to automatically identify clinical entities in electronic medical records (EMRs), is an important research direction of clinical text data mining and information extraction. The promotion of CNER can provide support for clinical decision making and medical knowledge base construction, which could then improve overall medical quality. Compared with English CNER, and due to the complexity of Chinese word segmentation and grammar, Chinese CNER was implemented later and is more challenging.

**Objective:**

With the development of distributed representation and deep learning, a series of models have been applied in Chinese CNER. Different from the English version, Chinese CNER is mainly divided into character-based and word-based methods that cannot make comprehensive use of EMR information and cannot solve the problem of ambiguity in word representation.

**Methods:**

In this paper, we propose a lattice long short-term memory (LSTM) model combined with a variant contextualized character representation and a conditional random field (CRF) layer for Chinese CNER: the Embeddings from Language Models (ELMo)-lattice-LSTM-CRF model. The lattice LSTM model can effectively utilize the information from characters and words in Chinese EMRs; in addition, the variant ELMo model uses Chinese characters as input instead of the character-encoding layer of the ELMo model, so as to learn domain-specific contextualized character embeddings.

**Results:**

We evaluated our method using two Chinese CNER datasets from the China Conference on Knowledge Graph and Semantic Computing (CCKS): the CCKS-2017 CNER dataset and the CCKS-2019 CNER dataset. We obtained F1 scores of 90.13% and 85.02% on the test sets of these two datasets, respectively.

**Conclusions:**

Our results show that our proposed method is effective in Chinese CNER. In addition, the results of our experiments show that variant contextualized character representations can significantly improve the performance of the model.

## Introduction

### Background

Electronic medical records (EMRs) are an important data resource to describe patients’ disease conditions or treatment processes. They are records written by clinicians using unstructured free text to describe medical activities for individual patients. By analyzing EMRs, a large amount of patient-related medical knowledge can be mined [1]. With the generation of a larger number of EMRs and the potential demand for medical information services and medical decision support, they have attracted much attention from researchers.

Clinical named entity recognition (CNER) aims to automatically identify clinical entities in EMRs and classify them into predefined categories, such as disease, image review, laboratory examination, operation, drug, and anatomy [2]. CNER is the key component of clinical text mining and EMR information extraction research and is used for clinical decision support in medical informatics [3]. At the same time, CNER can also provide support for disease diagnosis and medical knowledge base construction, so as to improve overall medical quality [4]. Compared with English CNER and due to the complexity of Chinese word segmentation and grammar, Chinese CNER was implemented later and is more challenging. As a public task, Chinese CNER has been introduced three times at the China Conference on Knowledge Graph and Semantic Computing (CCKS), from 2017 to 2019, in order to promote the information extraction of Chinese EMRs. In this paper, we conducted research and experiments with our Chinese CNER approach, based on the CCKS-2017 (Task 2) CNER dataset and the CCKS-2019 (Task 1) CNER dataset.

CNER is generally performed as a sequence tagging problem to identify and extract entity references related to clinical medicine. For the English CNER task, several neural network architectures have been proposed and achieved excellent performance; among them, the most widely used system is a combination of bidirectional long short-term memory (BiLSTM) and conditional random fields (CRFs) [5-7]. Ma and Hovy [8] presented the BiLSTM-convolutional neural network (CNN)-CRF model with CNN and achieved an approximately equal performance. Compared to named entity recognition (NER) in other fields, Chinese CNER is more challenging. Medical texts often use nonstandard abbreviations, or the same entity has multiple forms; for example, “奥沙利铂” (oxaliplatin) is the same as “奥沙利柏” (oxaliplatin) [9]. The more critical problem is that the Chinese grammatical structure is more complex than the English structure, and there is no natural word-segmentation boundary in Chinese, which may lead to word-segmentation error propagation in CNER [10]. In view of the dependence of Chinese word segmentation, Zhang and Yang [11] put forward an innovative lattice long short-term memory (LSTM) model for Chinese NER. Lattice LSTM is character based and effectively utilizes the corresponding potential word information, which is superior to character-based and word-based models in many Chinese general datasets.

Compared with statistical learning methods, which need to design or extract hand-crafted features based on domain-specific knowledge, deep learning methods usually use distributed representation as the input feature. Traditional pretrained character-embedding models, such as word2vec [12] and Global Vectors for Word Representation (GloVe) [13], train embedding based on their syntactic and semantic similarity in sentence-level contexts, but the training result is a context-independent character vector. In fact, a character may have completely different meanings in different contexts. For instance, in the sentence “考虑为腺癌，于5月30日给予TP方案化疗（紫杉醇240MG静脉滴注，顺铂90MG腹腔灌注），过程顺利，无明显副作用,” the meanings of both characters “顺” are different depending on their context. Reasonably, the two characters “顺” should have different vector representations. The Embeddings from Language Models (ELMo) [14] model, which provides deep contextualized word representations, allows the same word to have different vector representations in different sentences. The ELMo model was originally proposed for English text and generates specific English word vectors for each sentence, not character vectors. However, the lattice LSTM model is essentially based on Chinese characters; therefore, we modified the ELMo model to replace the character-encoding layer with domain-specific Chinese characters as input, so that the domain-specific ELMo embedding of Chinese characters was obtained.

In this paper, we propose a lattice LSTM model combined with a variant contextualized character representation and CRF layer for Chinese CNER. By taking advantage of the lattice LSTM structure, our approach can control the long-term state with the combination of word information to make full use of EMR information. Moreover, a variant ELMo model is projected into the lattice LSTM model to help it obtain contextual semantic information. Finally, a CRF layer is used to capture the dependencies between adjacent labels. We can summarize the main contributions of our work as follows:

1. We used the medical field texts to train domain-specific character embedding and word embedding; since traditional word embedding is difficult to use for capturing contextual semantics, the addition of the variant ELMo model can help the model combine the contextualized character representations on the basis of character information and potential word information.
2. This is the first time the variant ELMo embedding has been integrated into the lattice LSTM model and applied to Chinese CNER research. Compared with other prevalent models, it has achieved relatively competitive results with F1 scores of 90.13% and 85.02% on two Chinese CNER datasets, respectively.

### Prior Work

#### CNER

In the first research studies on CNER, rule-based methods [15] and dictionary-based methods [16] were the most common methods. For instance, Savova et al [17] and Zeng at al [18] combined manual rules and heuristic rules to identify medical entities with good results. Because of the grammatical complexity of Chinese clinical texts, rule-based methods need a lot of hand-crafted rules, which cannot identify enough entities and are difficult to transfer to other fields. Statistical learning algorithms are mainly based on single-word classification or sequence tagging, which can consider the tagging results of adjacent words jointly [19,20]; these algorithms include support vector machines (SVMs) [21], CRFs [22], and structured SVMs. Finkel et al [23] used CRF to establish an automatic annotation model for NER, which mainly considered the characteristics of words, prefixes, parts of speech sequences, and word morphologies. However, statistical learning methods rely heavily on complex feature engineering and resources for specific tasks. Collobert et al [24] took the lead in solving the NER problem with a neural model, and used the word embedding as the input feature. With the extensive application of deep learning in the field of natural language processing (NLP), various neural networks have been applied to sequence tagging tasks [25].

Systematic research on EMR entity recognition was initiated by i2b2 (Informatics for Integrating Biology and the Bedside) as a public evaluation task in 2010 [26]. This evaluation first classified EMR entities [27], mainly identifying three types of entities: medical problems, treatment, and examination. For Chinese CNER, Feng et al [28] first carried out CNER research on Chinese EMRs, using the CRF model and manually compiled dictionaries. In the Chinese CNER, the open dataset is extremely lacking, and only the CCKS evaluation tasks published the datasets; they were published three times, between 2017 and 2019. The BiLSTM-CRF model, with self-taught and active learning proposed by Xia and Wang [29], reached an F1 score of 88.98% on the CCKS-2017 CNER dataset. Since there is no clear word-boundary information in Chinese text, Chinese CNER systems can be generally divided into character-based and word-based methods. However, the character-based method may lose word-level information, while the word-based method suffers from word-segmentation error propagation.

#### Word Embedding

In general, the deep learning method uses word embedding trained from a large-scale unlabeled corpus as a model input instead of feature engineering. The most representative, pretrained word vectors—word2vec [12], GloVe [13], and a semisupervised learning method [30]—can capture fine-grained semantic and syntactic information from unlabeled text. Most of the pretrained word-embedding models are trained on the general corpus, and the semantic similarity measurement built for a general purpose is not effective in a specific field. In specific fields such as clinical text mining, there are many clinical entities and syntactic blocks that contain rich domain information, and the semantics of words are closely related to them; therefore, we need to use a specific corpus to train domain-specific embedding [31].

Most of the embedding models only produce context-independent representation for each word, so it is difficult to obtain contextual semantic information. Current research focuses on contextual vector representation; for example, context2vec [32] uses the LSTM model to encode context around a center word or some unsupervised language model [33]. Devlin et al [34] proposed a pretrained language model, Bidirectional Encoder Representations from Transformers (BERT), which achieved state-of-the-art results in many NLP tasks. This paper adopts the contextualized word-embedding (ie, ELMo) model introduced by Peters et al [14] and modifies it to adapt to Chinese characters.

## Methods

### Model

#### Overview

**Figure 1:**
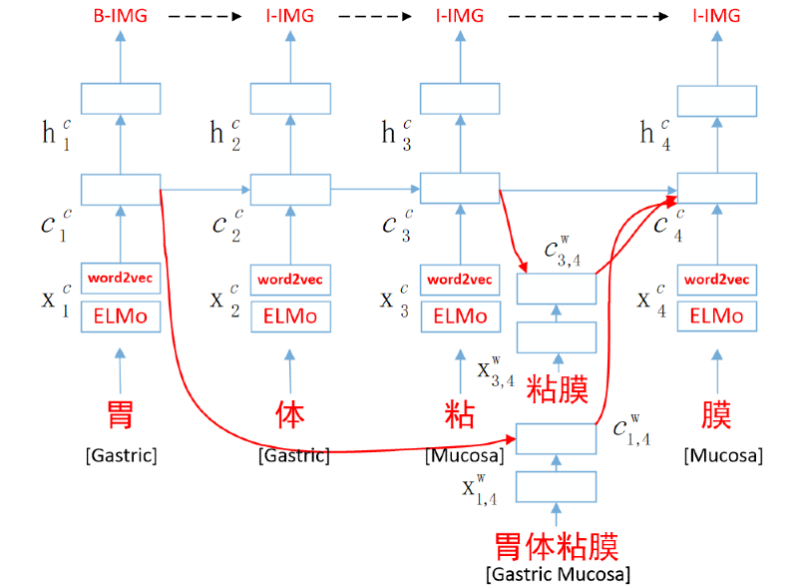
Architecture of the ELMo-lattice-LSTM-CRF model. B-IMG: beginning of image entity; c: cell memory; CRF: conditional random field; ELMo: Embeddings from Language Models; h: hidden state; I-IMG: inside of image entity; LSTM: long short-term memory; superscript c: character sign; superscript w: word sign; x: embedding of a character or word.

In this section, we propose the ELMo-lattice-LSTM-CRF model in detail; its architecture is shown in Figure 1. First, we concatenated the ELMo embedding and the word2vec embedding as the input of the character-embedding part of the lattice LSTM model. Second, embedding of the subsequence from lexicon *D* was used as the input of the word-embedding part. Finally, a CRF layer was used to predict the label probability. We illustrate these three parts of the ELMo-lattice-LSTM-CRF model with real clinical text (ie, “胃体粘膜” [gastric mucosa]) as an example.

#### Lattice LSTM

The lattice LSTM model can be regarded as an extension of the character-based method, which takes the addition of character embedding and weighted-word embedding as the input of the model. The input is a sequence of *m* characters as (*c*_1_*, c*_2_*,..., c_m_*), together with words that are obtained by matching the clinical text in lexicon *D*. We used the Gensim word2vec tool to train the unlabeled clinical corpus to obtain domain-specific character embedding and word embedding. This clinical corpus includes the CCKS-2017 CNER dataset, the CCKS-2019 CNER dataset, the unlabeled corpora provided by these two tasks, a health care and learning community [35], and the China National Knowledge Infrastructure (CNKI) medical abstracts [36], with a total of 526,631 sentences. In the known literature, there is no publicly available medical domain lexicon *D*, so we use the annotated entities in the Chinese CNER datasets provided by the CCKS-2017 and CCKS-2019 datasets and the dictionaries captured through open sources; finally, we built a medical terminology dictionary at a scale of about 23 kB. The term *w^d^_b,e_* denotes the subsequence of matching lexicon *D* in clinical text, beginning with character index *b* and ending at index *e*, as an example in Figure 1; the subsequence *w^d^_1,2_* is “胃体” (gastric), and *w^d^_1,4_* is “胃体黏膜” (gastric mucosa). The term *x^w^_b,e_* is the embedding of subsequence *w^d^_b,e_*. The character-level recurrent LSTM functions are shown below:

*f^c^_t_ = σ (W^ct^_f_ [h^c^_t–1_, x^c^_t_]) + b_f_***(1)**

*o^c^_t_ = σ (W^ct^_o_ [h^c^_t–1_, x^c^_t_]) + b_o_***(2)**

*i^c^_t_ = σ (W^ct^_i_ [h^c^_t–1_, x^c^_t_]) + b_i_***(3)**

(c^c^_t_)~ = tanh (W^ct^_c_[h^c^_t–1_, x^c^_t_]) + b_c_**(4)**

*c^c^_t_ = f ^c^_t_ × c^c^_t–1_ + i^c^_t_ × (c^c^_t_ )~***(5)**

*h^c^_t_ = o^c^_t_ × tanh (c^c^_t_)***(6)**

where *i^c^_t_*, *o^c^_t_*, *f^c^_t_*, and *c^c^_t_* represent input, output, forget gates, and the cell memory, respectively. *W* and *b* are model parameters and *σ ( )* denotes the sigmoid function.

A word cell *c^w^_b,e_*, which is calculated by the following formula, is used to represent the recurrent state of *x^w^_b,e_*:

*f^w^_b,e_ = σ (W^wt^_f_ [x^w^_b,e_, h^c^_b_]) + b_f_***(7)**

*i^w^_b,e_ = σ (W^wt^_i_ [x^w^_b,e_, h^c^_b_]) + b_i_***(8)**

*(c^w^_b,e_)~ = tanh (W^wt^_c_ [x^w^_b,e_, h^c^_b_]) + b_c_***(9)**

c^w^_b,e_ = f^w^_b,e_ × c^c^_b_ + i^w^_b,e_ × (c^w^_b,e_)~ **(10)**

where *i^w^_b,e_* is the input gate and *f^w^_b,e_* is the forget gate. Compared with the standard LSTM model, there is no output gate for word units, since label prediction is only on the character sequence.

At each time step, multiple information *c^w^_b,e_* flows into *c^c^_t_* through recurrent paths. Take the previous clinical text as an example: the input resources for *c^c^_4_* include *x^c^_4_* (“膜” [mucosa]), *c^w^_3,4_* (“粘膜” [mucosa]), and *c^w^_1,4_* (“胃体黏膜” [gastric mucosa]). We add all *c^w^_b,e_* with weights *b ~∈(b ~|i^w^_b ~,e_ ∈D)* to *c^c^_e_*; an additional gate *i^c^_b,e_* controls the contribution of each subsequence into *c^c^_e_*:

*i^c^_b,e_ = σ ([x^c^_e_, c^w^_b,e_]) + b^l^***(11)**

The function for calculating cell values *c^c^_t_* becomes equation 12. Among them, the gate values *i^c^_b,t_* and *i^c^_j_* are normalized (sum to 1) to *α^c^_b,t_* and *α^c^_t_*:

*c^c^_t_ = ∑_weights_α^c^_b,t_ × c^w^_b,t_ + α^c^_t_ × (c^c^_t_)~***(12)**

The final hidden vectors *h^c^_t_* are still calculated according to equation 6. According to the above deduction, we find that the lattice LSTM model can focus on relevant words dynamically during NER labeling and can make comprehensive use of the character information and word information of clinical text.

#### ELMo

Unlike most widely used, pretrained word-embedding models, ELMo [14] word representations are calculated by the entire input sentence. The sentence first passes through a convolutional character-encoding layer; it is then sent to the two-layer bidirectional language model (BiLM) layer, and the resulting vector is sent to the scalar mixer layer to get the ELMo embedding. Specifically, given a sequence of *N* tokens (*t*_1_, *t*_2_,..., *t_N_*), a BiLM computes and combines the current tokens’ *t_k_* probabilities in both the forward and backward directions. Its goal is to maximize the following likelihood values:

*∑^N^_k=1_ (logp (t_k_|t_1_,…,t_k–1_; θ_x_, θ_LSTM_(right),θ_s_) + logp (t_k_|t_k+1_,…,t_N_; θ_x_, θ_LSTM_(left), θ_s_))***(13)**

Where *θ_x_, θ_s_, θ_LSTM_(right),* and *θ_LSTM_(left)* are the token representation, the Softmax layer, and the forward- and backward-direction LSTM parameters, respectively.

For each token *t_k_*, an L-layer BiLM calculates a set of 2L+1 representations as follows:

*R_k_ = {X^LM^_k_,h^LM^_k,j_(right), h^LM^_k,j_(left)|j=1,…,L} = {h^LM^_k,j_|j=0,…,L}***(14)**

Where *h^LM^_k,0_ is* the token layer and *h^LM^_k,j_ = [h^LM^_k,j_(right); h^LM^_k,j_(left)]* for each BiLSTM layer.

For these representations, the paper makes a scalar mixer with the following formula:

*ELMo^task^_k_ = E(R_k_; θ^task^) = ϒ^task^∑^L^_j=0_ s^task^_j_ h^LM^_k,j_***(15)**

Here, *s^task^* is the Softmax-normalized weight, and the scalar parameter *ϒ^task^* is used to scale the whole ELMo vector.

In the specific application, the model is pretrained on a large-scale unlabeled corpus. After the model is trained, a new sentence is input to get the contextualized ELMo embedding of each word in the current context. The original ELMo model was proposed for English text, and English words are divided into English character sequences as input, resulting in ELMo embedding of English words. Che et al [37] applied ELMo to multiple languages, including Chinese. They used the Chinese word-segmentation tools to segment text into words, and then used the ELMo model to obtain the contextualized word embedding.

**Figure 2:**
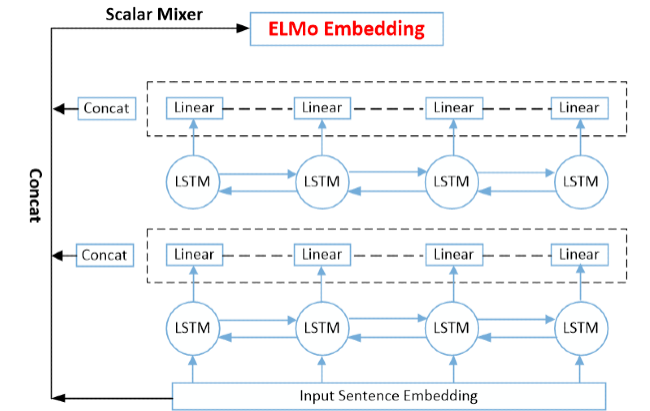
Architecture of the variant Embeddings from Language Models (ELMo) model. concat: concatenate; LSTM: long short-term memory.

In the method we proposed, in addition to the standard input of the lattice LSTM model, we integrated the domain-specific, pretrained ELMo embedding of Chinese characters as one of the input features. For obtaining the ELMo embedding of Chinese single characters, we used space to cut the corpus into single-character forms. Then, we modified the ELMo model; the architecture of the variant ELMo model is shown in Figure 2. We removed the convoluted character-encoding layer, and the embedding of Chinese characters was used as the input for training, with the dimension of character embedding set to 100. The input-sentence embedding was sent to the two-layer BiLSTM layer and two-layer representations were obtained. In the original work, the hidden size of the LSTM unit was set to be larger, and the dimension needed to be mapped to 512 through the linear layer, so that the output vector dimension of each character by each BiLSTM layer would be 1024. In our approach, we also modified the linear layer and mapped the hidden size of the LSTM cell to 50 through the linear layer; the output vector dimension of each token by each BiLSTM layer become 100. We then concatenated the input-sentence embedding and two-layer representations of the two-layer BiLSTM; the resulting vector was sent to the scalar mixer layer. Finally, pretrained ELMo embedding of Chinese characters was obtained by equation 15. At the pretrained stage of the ELMo model, we used the same unlabeled clinical corpus as done with the training-character embedding. In the application, a clinical sentence was sent into the pretrained ELMo model, so the ELMo embedding was obtained.

#### CRF

A CRF layer is used on hidden vectors (*h^c^*_1_, *h^c^*_2_,..., *h^c^_t_*). The CRF decodes *h^c^_t_* into *k*-dimensional vectors, which denote label prediction probabilities. The score of the prediction sequence *y* = (*y*_1_, *y*_2_, *y*_3_,..., *y_n_*) is computed by the following formula:

*S(X,y) = ∑^n^_i=1_ p_i,j_ +∑^n+1^_i=1_ A_y(i–1)_,_y(i)_***(16)**

where *p_i,j_* denotes the probability of label *j* for word *i*, *A* represents the tagging transition matrix, and *A_i,j_* represents the score of the transition from label *i* to *j*.

Finally, the conditional probability *P(y|X)* is calculated as follows:

*P(y|X) = exp(score(X,y)) / ∑_y’_exp(score(X,y’))***(17)**

where *X* = (*x*_1_, *x*_2_, *x*_3_,..., *x_n_*), which represents the character sequence input.

### Model Implementation

In order to evaluate the performance of our approach, we implemented a series of basic models for comparison, as listed below:

1. Char-BiLSTM-CRF. This is a character (char)-based baseline model [29] without word segmentation; domain-specific character embedding was used as input. The pretrained character embedding was trained using the self-constructed clinical corpus mentioned in the Lattice LSTM section, and its dimension is 100.
2. BERT-BiLSTM-CRF. We used the pretrained RoBERTa_middle embedding model [38,39]—an improved version of BERT—as the input into the BiLSTM layer instead of the character embedding.
3. Word-BiLSTM-CRF. This is a word-based baseline model with reference to Wu et al [40]. We used the jieba segmentor [41], which includes the lexicon D, to segment the corpus. The Chinese word embedding in the medical field was trained by the word2vec tool, and the dimension was set to 100.
4. Word-BiLSTM-CRF (char CNN). On the basis of the word-based baseline model, the character-level embedding of words or subsequences was introduced [8]. The Chinese character in a word or subsequence is the smallest semantic unit, which carries certain information. The dimension of character-level embedding was set to 50, and the embedding lookup table was randomly initialized. The final state of character-level embedding was obtained by a CNN model; it was then concatenated with the word embedding to obtain the distributed representation of the word subsequence.
5. Word-BiLSTM-CRF (char LSTM). Similar to the above structure, the difference is that the LSTM model was used to encode character-level embedding [42].
6. ELMo-lattice-LSTM-CRF. This structure was our proposed method. The pretrained word2vec character embedding was combined with the medical field, pretrained, ELMo character embedding as the character part input of the model. The word subsequence was obtained by matching sentences in lexicon D, and its embedding was the same as that of the word-based baseline model.

### Parameter Settings

In this study, we cut sentences into character sequences and limited the length to no more than 200. The BIO (beginning, inside, outside) schema was taken to annotate the entity. As mentioned earlier, the pretrained character embedding, word embedding from lexicon *D*, and ELMo embedding were all 100-dimensional vectors. The number of layers of LSTM was 1 and the hidden size was 200. We set the epoch to 10, the batch size to 1, and the dropout rate was 0.5. We adopted categorical cross-entropy to compute the loss function. A stochastic gradient descent optimizer, with a learning of 0.015 and decay rate of 0.05, was used to update parameters. The detailed settings of hyperparameters are shown in Table 1; similar parameters were used in other baseline models. On two Chinese CNER datasets, we used the same parameters, embedding, and lexicon to evaluate our method. Finally, we used the deep learning framework pytorch [43] to implement our model.

**Table 1:** Hyperparameter settings of the proposed approach.

Parameter	Value
Character-embedding size	100
Embeddings from Language Models (ELMo) embedding size	100
Word-embedding size	100
Dropout rate	0.5
Long short-term memory (LSTM) hidden size	200
LSTM layer	1
Learning rate	0.015
Learning rate decay	0.05
Epoch	10
Batch size	1

## Results

### Dataset and Evaluation Metrics

We conducted experiments based on two datasets, both of which were processed to delete privacy in the annotation phase. The first dataset was the CCKS-2017 CNER dataset, which contains 1596 labeled EMRs with five categories of clinical entities, including diseases, symptoms, exams, treatments, and body parts. We divided the dataset into two parts: 1198 EMRs were taken as a training set and 398 EMRs were taken as test set. Sequences that are too long will lead to the deterioration of model performance, so punctuation was used to split EMRs into sentences [11]. Therefore, the training set contained 7906 sentences and the test set contained 2118 sentences. The detailed distribution of the count of different types of entities is shown in Table 2.

The second dataset was the CCKS-2019 CNER dataset, which contains 1000 labeled EMRs. We divided the dataset into 900 training EMRs (5872 sentences) and 100 test EMRs (612 sentences). There were six categories of clinical entities in the dataset: disease, image, laboratory, operation, drug, and anatomy. The detailed distribution of the count of different types of entities is shown in Table 3.

In this paper, we used standard evaluation metrics, such as precision, recall, and F1 scores, to evaluate model performance. Meanwhile, the evaluation metrics were strict, which requires that the true label and prediction label have exactly the same entity name, same boundary, and same entity type.

**Table 2:** The distribution of entities in the China Conference on Knowledge Graph and Semantic Computing (CCKS)-2017 clinical named entity recognition (CNER) dataset.

Dataset	Number of entities in each category					
	Sentence	Disease	Symptom	Exam	Treatment	Body part
Training set	7906	722	7831	9546	1048	10,719
Test set	2118	553	2311	3143	465	3021

**Table 3:** The distribution of entities in the China Conference on Knowledge Graph and Semantic Computing (CCKS)-2019 clinical named entity recognition (CNER) dataset.

Dataset	Number of entities in each category						
	Sentence	Disease	Image	Laboratory	Operation	Drug	Anatomy
Training set	5872	3755	940	1167	932	1586	7524
Test set	612	362	34	37	116	242	898

### Experiments Results

In order to get convincing experimental results, we ran each model five times and calculated the average precision, recall, and F1 scores as the final results. Table 4 shows the results of various models with different architectures on the test set of two Chinese CNER datasets.

**Table 4:** Results of various models with different architectures on two datasets.

Model	CCKS^a^-2017 CNER^b^ dataset			CCKS-2019 CNER dataset		
	Precision, %	Recall, %	F1 score, %	Precision, %	Recall, %	F1 score, %
Char^c^-BiLSTM^d^-CRF^e^ (baseline)	88.86	86.78	87.81	81.67	80.01	80.83
BERT^f^-BiLSTM-CRF	87.42	86.37	86.89	79.58	80.67	80.12
Word-BiLSTM-CRF (baseline)	85.87	86.33	86.10	79.63	80.07	79.85
Word-BiLSTM-CRF (char CNN^g^)	88.23	86.90	87.56	82.69	81.72	82.20
Word-BiLSTM-CRF (char LSTM^h^)	89.86	87.34	88.58	83.58	82.21	82.89
ELMo^i^-lattice-LSTM-CRF	*90.20* ^j^	*90.06*	*90.13*	*84.69*	*85.35*	*85.02*

^a^CCKS: China Conference on Knowledge Graph and Semantic Computing.

^b^CNER: clinical named entity recognition.

^c^char: character.

^d^BiLSTM: bidirectional long short-term memory.

^e^CRF: conditional random field.

^f^BERT: Bidirectional Encoder Representations from Transformers.

^g^CNN: convolutional neural network.

^h^LSTM: long short-term memory.

^i^ELMo: Embeddings from Language Models.

^j^The best experimental results are italicized.

We observed that the character-based baseline model was better than the BERT-BiLSTM-CRF model, which is also character based and used the state-of-the-art pretrained BERT embedding. The main reason for this result is that BERT embedding was trained on the general field corpus rather than on the domain-specific corpus, which reflects the complexity of Chinese clinical texts. The character-based baseline model was better than the word-based baseline model as a whole, which shows that the character-based method can make better use of medical text information in Chinese CNER tasks.

It can be seen from the table that the word-BiLSTM-CRF (char LSTM) model outperformed the character-based and word-based baseline models and obtained competitive F1 scores of 88.58% and 82.89% on two datasets, respectively. This shows that the introduction of character-level embedding in the word-based method can make relatively full use of character and word information and can effectively improve the performance of the model. In addition, we also observed that the LSTM model captured the character-level semantic information of words better than did the CNN model.

From the results, we observed that the ELMo-lattice-LSTM-CRF model we proposed, which integrates lattice LSTM structure and variant pretrained ELMo embedding, achieved excellent results compared with the other models on both Chinese CNER datasets. This was seen with the F1 scores that reached 90.13% on the CCKS-2017 CNER dataset and 85.02% on the CCKS-2019 CNER dataset. Compared with the word-BiLSTM-CRF (char LSTM) model, the F1 scores of our method on both datasets were significantly improved by 1.55% and 2.57%, respectively. Table 5 shows the results of our method compared with previous representative systems on these two datasets [42,44,45].

The system in the first line [42] also used both Chinese character embedding and word embedding as feature representations, and an external health domain lexicon was adopted, which achieved an F1 score of 87.95% on the CCKS-2017 CNER dataset. The system in the second line [44] was similar to that in this paper. It adopted a lattice LSTM structure and used an adversarial training approach to improve the performance of the model; it achieved a good result, with an F1 score of 89.64%. The results show that our method surpassed these two systems by 2.18% and 0.49%, respectively. For the CCKS-2019 CNER dataset, Li et al [45] achieved the top performance by adopting the method of transfer learning and ensemble; our method obtained a similar score. By comparing our method with the previous models, the effectiveness of our method is evident.

**Table 5:** Comparative results between our approach and previous systems on two datasets.

Model	CCKS^a^-2017 CNER^b^ dataset			CCKS-2019 CNER dataset		
	Precision, %	Recall, %	F1 score, %	Precision, %	Recall, %	F1 score, %
Recurrent neural network (char^c^-word) [42]	—^d^	—	87.95	—	—	—
AT^e^-lattice-LSTM^f^-CRF^g^ [44]	88.98	90.28	89.64	—	—	—
FS^h^-TL^i^ (ensemble) [45]	—	—	—	—	—	*85.16* ^j^
Our approach	*90.20*	90.06	*90.13*	84.69	85.35	85.02

^a^CCKS: China Conference on Knowledge Graph and Semantic Computing.

^b^CNER: clinical named entity recognition.

^c^char: character.

^d^Data not available.

^e^AT: adversarial training.

^f^LSTM: long short-term memory.

^g^CRF: conditional random field.

^h^FS: fully shared.

^i^TL: transfer learning.

^j^The best experimental results are italicized.

## Discussion

### Overview

By comparing the experimental results, we notice that our method has excellent performance on the Chinese CNER task, which surpassed the character-based and word-based methods. In the future, we will conduct ablation experiments to further explore the influence of the lattice LSTM structure and ELMo embedding on the model performance.

### Dataset Analysis

**Figure 3:**
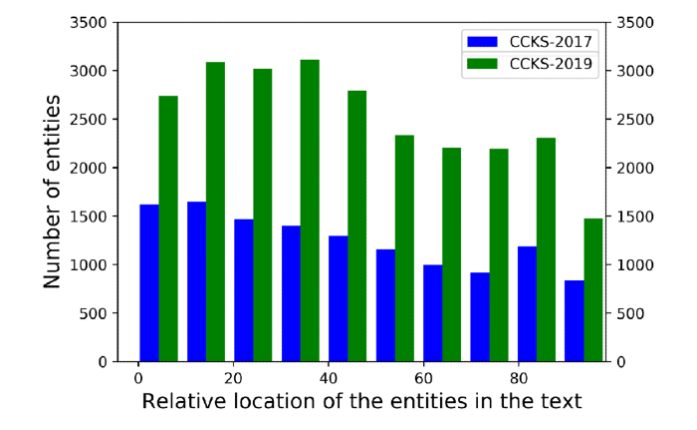
Distribution of relative locations of entities in two Chinese clinical named entity recognition (CNER) datasets. CCKS: China Conference on Knowledge Graph and Semantic Computing.

First, we analyzed the two Chinese CNER datasets. Figure 3 shows the distribution of the relative locations of clinical entities in the training set of the two datasets.

From the figure, we can intuitively observe that the distribution of entity locations in the two datasets is similar and relatively uniform; however, the distribution of entities from the CCKS-2019 CNER dataset is obviously more sparse than that of the CCKS-2017 CNER dataset. This indicates that the CCKS-2019 dataset labels were relatively unbalanced and there were more *outside* labels, which explains the reason why the results from the same models using CCKS-2017 CNER dataset were superior to those using the CCKS-2019 CNER dataset. Meanwhile, Tables 2 and 3 showed that there were very few image entities and laboratory entities in the test set—34 and 37, respectively—compared with the training set from the CCKS-2019 CNER dataset. This means that the distribution of labels in the test set and training set from the CCKS-2019 CNER dataset was quite different, which is another reason for the weaker performance by the model when using the CCKS-2019 CNER dataset.

### Effectiveness of the Lattice LSTM Model

The comparison of the results of the standard lattice LSTM model and the character-based and word-based methods from using the two datasets is shown in Table 6. From the table, we observe that the performance of the standard lattice LSTM model surpassed that of the char-BiLSTM-CRF and word-BiLSTM-CRF (char LSTM) models. Compared with the better-performing word-BiLSTM-CRF (char LSTM) model, the performance of the model using the lattice LSTM on CCKS-2017 CNER dataset improved by 0.84%; the performance on the CCKS-2019 CNER dataset significantly improved by 1.29%. Although the word-BiLSTM-CRF (char LSTM) and lattice LSTM models used the same word embedding and lexicon, the word-BiLSTM-CRF (char LSTM) model first uses the lexicon for word segmentation, which imposes a hard restriction on the use of its subsequences, while the lattice LSTM model is free to consider lexicon words. This provides evidence that the lattice LSTM model can dynamically integrate potential word information, is superior to the character-based and word-based methods, and can achieve excellent performance in solving the Chinese CNER problem.

**Table 6:** Comparison of results between character-based or word-based methods and the lattice long short-term memory (LSTM) model on two datasets.

Model	CCKS^a^-2017 CNER^b^ dataset				CCKS-2019 CNER dataset		
	Precision, %	Recall, %	F1 score, %	Precision, %		Recall, %	F1 score, %
Char^c^-BiLSTM^d^-CRF^e^ (baseline)	88.86	86.78	87.81	81.67		80.01	80.83
Word-BiLSTM-CRF (char LSTM^f^)	*89.86* ^g^	87.34	88.58	83.58		82.21	82.89
Lattice-LSTM-CRF	89.66	*89.18*	*89.42*	*85.11*		*83.27*	*84.18*

^a^CCKS: China Conference on Knowledge Graph and Semantic Computing.

^b^CNER: clinical named entity recognition.

^c^char: character.

^d^BiLSTM: bidirectional long short-term memory.

^e^CRF: conditional random field.

^f^LSTM: long short-term memory.

^g^The best experimental results are italicized.

### Effectiveness of ELMo Embedding

Table 7 shows the comparative results of different types of character embedding that were added to the lattice LSTM model using the two CNER datasets. The first line is the standard lattice LSTM model, and the second line is an embedding with equal dimensions and random initialization. It can be seen that there were slight improvements on both datasets, which may be due to the increase in parameters. In the third line, the character embedding trained by the GloVe tool [13] was added, and the F1 scores on the two datasets reached 89.70% and 84.62%, respectively, which shows that the addition of domain-specific character embedding is effective. The performance of the ELMo-lattice-LSTM-CRF (ML [many languages]) model, with pretrained ELMo representation for multiple languages [37,46], was slightly reduced compared to the standard lattice-LSTM-CRF model. This is likely because the pretrained ML model was trained on the general field corpus, so there was the problem of semantic inaccuracy.

**Table 7:** Comparison of different types of character embedding added to the lattice long short-term memory (LSTM) model using two clinical named entity recognition (CNER) datasets.

Model	CCKS^a^-2017 CNER^b^ dataset			CCKS-2019 CNER dataset		
	Precision, %	Recall, %	F1 score, %	Precision, %	Recall, %	F1 score, %
Lattice-LSTM^c^-CRF^d^	89.66	89.18	89.42	85.11	83.27	84.18
Random-lattice-LSTM-CRF	88.79	*90.32* ^e^	89.55	85.10	83.65	84.37
GloVe^f^-lattice-LSTM-CRF	89.63	89.77	89.70	*85.32*	83.90	84.62
ELMo^g^-lattice-LSTM-CRF (ML^h^)	89.90	88.69	89.29	82.23	84.09	83.15
ELMo-lattice-LSTM-CRF	*90.20*	90.06	*90.13*	84.69	*85.35*	*85.02*

^a^CCKS: China Conference on Knowledge Graph and Semantic Computing.

^b^CNER: clinical named entity recognition.

^c^LSTM: long short-term memory.

^d^CRF: conditional random field.

^e^The best experimental results are italicized.

^f^GloVe: Global Vectors for Word Representation.

^g^ELMo: Embeddings from Language Models.

^h^ML: many languages.

The experimental results show that our proposed method was the best among all the methods, and it exceeded the standard lattice LSTM model by 0.71% and 0.84% on two datasets, respectively. These results demonstrate that the pretrained ELMo embedding trained on the medical corpus can further improve the performance of the model. After adding the pretrained ELMo embedding, the model used character information and weighted potential word information in sentences through the lattice LSTM structure; the model also obtained the domain-specific contextualized character representations, so as to obtain the rich semantic information of the EMRs, which is conducive to improving the performance of the model in the Chinese CNER task.

### Error Analysis

We carried out error analysis on each entity category and on the reasons for misclassification. As shown in Table 8, we compared the results of our method with those of the char-BiLSTM-CRF model and the word-BiLSTM-CRF (char LSTM) model with respect to various entity categories: disease, image, laboratory, operation, drug, and anatomy. Since the distribution of results was similar, only the results of the CCKS-2019 CNER dataset are used for illustration.

**Table 8:** Comparison of the results regarding each entity category when using the China Conference on Knowledge Graph and Semantic Computing (CCKS)-2019 clinical named entity recognition (CNER) dataset.

Model	F1 scores for each entity category, %						
	Disease	Image	Laboratory	Operation	Drug	Anatomy	All
Char^a^-BiLSTM^b^-CRF^c^	80.23	77.75	74.41	*83.61* ^d^	88.74	80.25	80.83
Word-BiLSTM-CRF (char LSTM^e^)	81.45	80.56	77.41	81.54	91.86	*84.56*	82.89
ELMo^f^-lattice-LSTM-CRF	*83.66*	*85.23*	*78.28*	82.12	*97.05*	83.79	*85.02*

^a^char: character.

^b^BiLSTM: bidirectional long short-term memory.

^c^CRF: conditional random field.

^d^The best experimental results are italicized.

^e^LSTM: long short-term memory.

^f^ELMo: Embeddings from Language Models.

From the table, our method showed a significant improvement regarding image and drug entities, with F1 scores 4.67% and 5.19% higher than the previous best results; in particular, the F1 score for the drug entity reached 97.05%. Through analysis, we determined that the improvement of image entities was mainly due to the fact that image entities are mostly compound words in Chinese CNER, such as “心脏彩超” (color Doppler ultrasound of the heart), “腹部彩超” (color Doppler ultrasound of the abdomen), and “肝脏彩超” (color Doppler ultrasound of the liver). For instance, “心脏彩超” is often divided into two parts: the anatomy entity “心脏” (heart) and the image entity “彩超” (color Doppler ultrasound). In the drug entity, single characters in terms such as “奥沙利铂” (oxaliplatin) and “希罗达” (Xeloda) are almost meaningless or even interfere with semantic understanding. Lattice LSTM improves the accuracy by constructing a medical domain lexicon and dynamically integrating word information. However, we noticed that all the methods did not perform well regarding the laboratory entity. This may be because laboratory entities are more complex than other entity types, in which mixed representations occur more often, such as “ca74-2,” “间接coombs试验” (indirect Coombs test), and “g6pd活性试验” (glucose-6-phosphate dehydrogenase [G6PD] activity test); in addition, entities can be too short, such as “氯” (chlorine), “hb,” and “ph.” This is still a great challenge for the research of Chinese CNER; it is also the direction in which future research is heading.

### Conclusions

By introducing the lattice LSTM model and a variant ELMo language model, this paper proposes a new Chinese CNER deep learning method. Our approach allows the model to coordinate the use of the character information and potential word information and takes advantage of contextualized character presentations, so as to make full use of EMR information. Finally, we used the CRF layer to capture the dependency between adjacent labels. We constructed a series of experiments on two Chinese CNER datasets to evaluate the performance of the model. The results showed that the ELMo-lattice-LSTM-CRF model that we proposed achieved excellent results, with F1 scores of 90.13% and 85.02% on the two datasets, respectively, which exceeded the performance of the standard lattice-LSTM-CRF model and achieved a competitive system. Overall, the results show that our approach for Chinese CNER is effective and can be used in future research. In future work, we will further generalize our model to improve its applicability and apply it to other small datasets through transfer learning methods.

## Abbreviations

BERT: Bidirectional Encoder Representations from Transformers
BiLM: bidirectional language model
BiLSTM: bidirectional long short-term memory
BIO: beginning, inside, outside
CCKS: China Conference on Knowledge Graph and Semantic Computing
char: character
CNER: clinical named entity recognition
CNN: convolutional neural network
CRF: conditional random field
ELMo: Embeddings from Language Models
EMR: electronic medical record
G6PD: glucose-6-phosphate dehydrogenase
GloVe: Global Vectors for Word Representation
i2b2: Informatics for Integrating Biology and the Bedside
LSTM: long short-term memory
ML: many languages
NER: named entity recognition
NLP: natural language processing
SVM: support vector machine
